# Severe Macular Commotio Retinae Following a Fall from a Horse in a Pediatric Patient

**DOI:** 10.3390/pediatric18030065

**Published:** 2026-05-02

**Authors:** Bogumiła Wójcik-Niklewska, Zofia Oliwa, Karina Dzięcioł, Adrian Smędowski

**Affiliations:** 1 Department of Pediatric Ophthalmology, Faculty of Medical Sciences in Katowice, Medical University of Silesia, 40-055 Katowice, Poland; asmedowski@sum.edu.pl; 2 Department of Pediatric Ophthalmology, Professor Kornel Gibiński University Hospital Center, Medical University of Silesia, 40-514 Katowice, Poland; 3 Students’ Scientific Society, Department of Ophthalmology, Faculty of Medical Sciences in Katowice, Medical University of Silesia, 40-752 Katowice, Poland; zofiaoliwa2002@gmail.com (Z.O.); karina.dzieciol@o2.pl (K.D.); 4 GlaucoTech Co., 40-282 Katowice, Poland

**Keywords:** commotio retinae, blunt ocular trauma, children, equestrian accident, retina

## Abstract

Background and Clinical Significance: Blunt ocular trauma is a significant but often underestimated cause of visual impairment, particularly among adolescents involved in high-risk activities such as horseback riding. While most equestrian injuries affect the head and extremities, ocular trauma, especially commotio retinae, can result in severe visual complications. Case Presentation: We report the case of a 15-year-old girl who sustained blunt ocular trauma to the left eye following a fall from a horse and presented with decreased visual acuity. Multimodal imaging revealed outer retinal abnormalities on spectral-domain optical coherence tomography (OCT), including ellipsoid zone irregularities. Early-phase fluorescein angiography showed central hypofluorescence in the foveal region with surrounding mild mottled hyperfluorescence, without clear vascular abnormalities. Fundus photography demonstrated subtle macular changes. Visual acuity improved significantly following treatment, with partial resolution of macular changes, although mild outer retinal irregularities persisted on follow-up imaging. Conclusions: These findings underscore the importance of early ophthalmic evaluation and advanced retinal imaging in blunt ocular trauma. Given the high risk of visual injury during equestrian activities, especially in pediatric and adolescent populations, preventive strategies such as mandatory helmet use and rider education are essential. Implementation of standardized follow-up protocols is also recommended to monitor long-term retinal changes in patients with traumatic maculopathy.

## 1. Introduction and Clinical Significance

Commotio retinae is a retinal condition caused by blunt ocular trauma, characterized by transient retinal whitening resulting from disruption of the photoreceptor outer segments and retinal pigment epithelium [1]. It is commonly observed after sports-related injuries, physical assaults, or accidental trauma. Although it typically follows a benign and self-limiting course, macular involvement may lead to significant visual impairment [2]. In pediatric patients, ocular trauma represents a major cause of preventable visual morbidity, and posterior segment injuries may be underrecognized due to delayed symptom reporting or limited cooperation during examination [3].

Macular edema is an uncommon but clinically relevant complication of commotio retinae, suggesting more severe retinal involvement and potentially affecting visual prognosis. Advances in retinal imaging, particularly optical coherence tomography (OCT), have improved the detection of subtle structural changes in the macula, enabling more accurate diagnosis and monitoring of traumatic retinal conditions [4]. However, reports describing commotio retinae with associated macular edema in children remain limited.

Equestrian activities are associated with a high risk of injury, particularly in pediatric and adolescent populations, and falls from horses can generate substantial blunt force even in the absence of direct ocular impact [5]. Despite this, detailed descriptions of posterior segment involvement in such cases are relatively scarce.

We present a case of a pediatric patient with commotio retinae and associated macular involvement following a fall from a horse, highlighting the role of multimodal imaging in the assessment and follow-up of traumatic retinal changes.

## 2. Case Presentation

A 15-year-old girl presented to the Ophthalmology Department on the day of injury with decreased visual acuity in the left eye following a fall from a horse associated with blunt trauma. A definite direct impact to the globe could not be established, and an indirect mechanism of injury cannot be excluded.

At presentation, corrected distance visual acuity was 1.0 in the right eye and 0.2 in the left eye. Intraocular pressure was within normal limits in both eyes. Slit-lamp examination revealed corneal scarring and opacities in the left eye, as well as a scar of the upper eyelid in the right eye. Fundus examination of the left eye demonstrated features consistent with commotio retinae involving both the central and peripheral retina, while the right eye was unremarkable. Automated perimetry showed no visual field defects in either eye.

Ocular ultrasonography of the left eye revealed hyperreflective echoes within the vitreous cavity and a partial posterior vitreous detachment, predominantly in the superior region.

**Figure 1:**
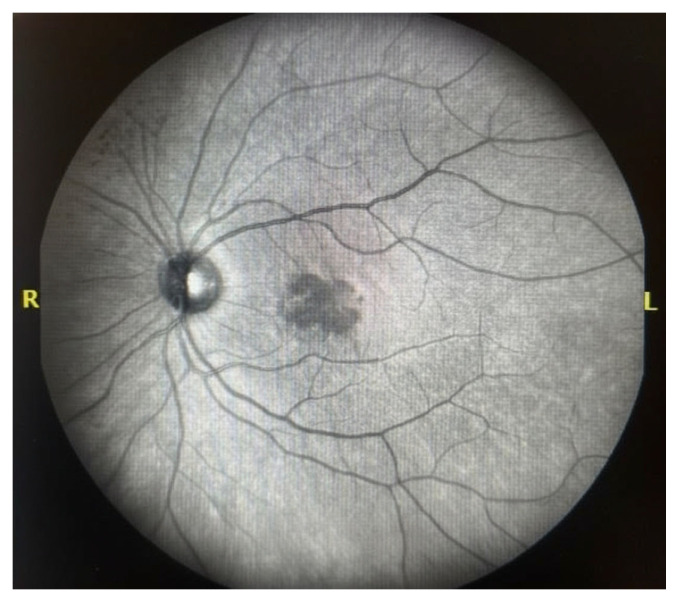
Early-phase fluorescein angiography of the left eye showing central hypofluorescence in the foveal region with surrounding mild mottled hyperfluorescence.

Early-phase fluorescein angiography demonstrated central hypofluorescence in the foveal region surrounded by mild mottled hyperfluorescence (Figure 1). The hypofluorescent appearance may reflect masking by overlying retinal changes.

**Figure 2:**
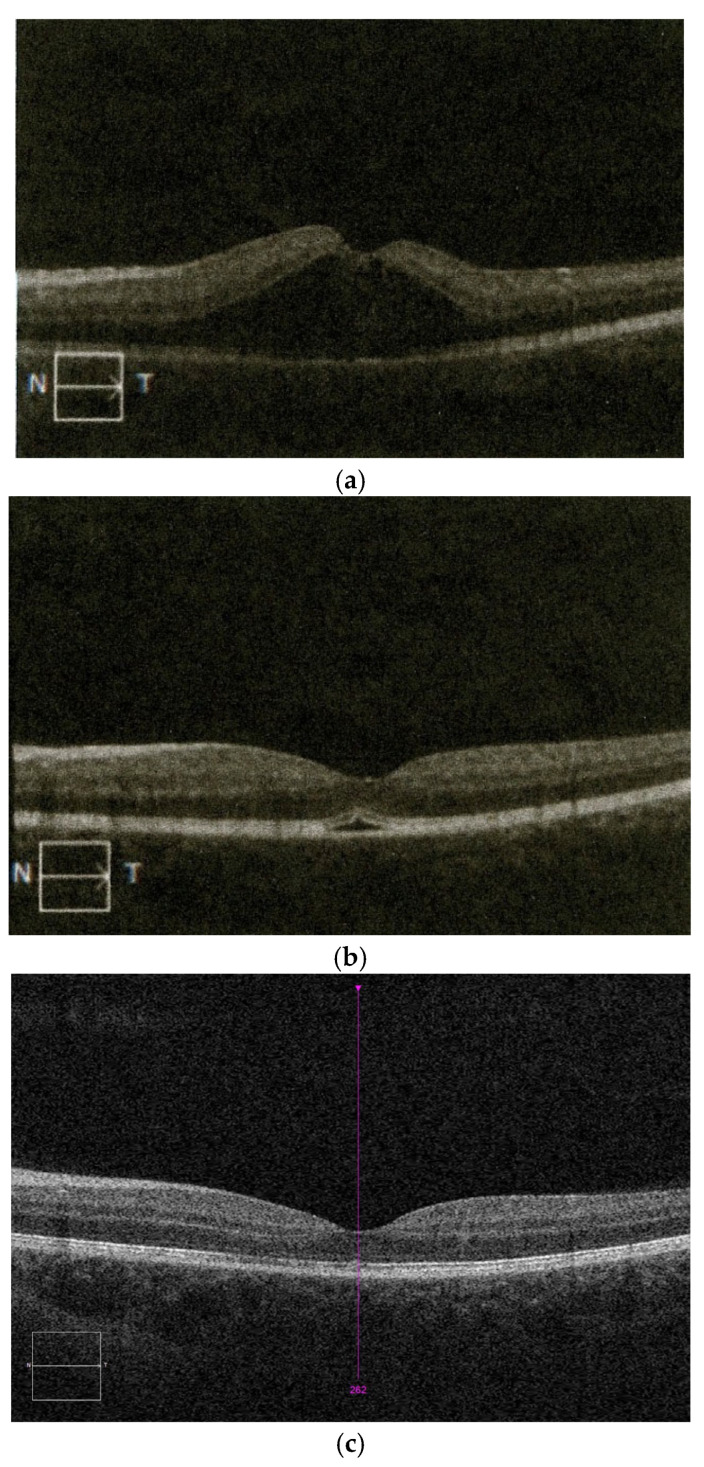
Spectral-domain optical coherence tomography (SD-OCT) images of the left eye showing dynamic macular changes: (**a**) on the day of injury—central foveal elevation; (**b**) one day after trauma—partial reduction in foveal elevation with persistent outer retinal irregularities; (**c**) three weeks after trauma—largely restored foveal architecture with subtle outer retinal abnormalities.

Further evaluation with spectral-domain optical coherence tomography (SD-OCT) revealed dynamic structural changes over time (Figure 2). On the day of injury (Figure 2a), OCT demonstrated a central foveal elevation suggestive of neurosensory detachment, with associated abnormalities of the outer retinal layers. On the following day (Figure 2b), a partial reduction in the foveal elevation was observed, although irregularities of the outer retinal layers persisted. Three weeks after trauma (Figure 2c), the foveal architecture appeared largely restored, although subtle outer retinal abnormalities remained.

**Figure 3:**
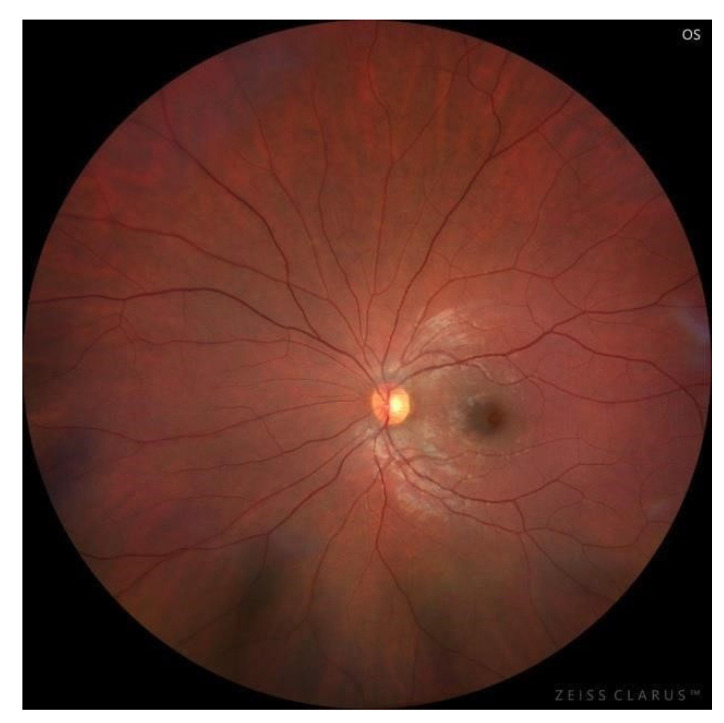
Widefield fundus photograph of the left eye showing a normal optic disc and retinal vasculature, with subtle discoloration in the macular region, absence of the foveal reflex, and mild alteration of the foveal contour.

Widefield color fundus imaging of the left eye (Figure 3) revealed a normal optic disc with sharp margins and normal retinal vasculature. The macula showed subtle foveal irregularity with absence of the foveal light reflex and mild alteration of the foveal contour.

Given the reduced visual acuity at presentation and the presence of acute post-traumatic macular involvement on multimodal imaging, in-hospital treatment was initiated with the aim of limiting secondary inflammatory response and supporting retinal recovery. Systemic methylprednisolone was administered, and topical nepafenac was prescribed as adjunctive anti-inflammatory therapy. Following treatment, visual acuity in the left eye improved significantly to 0.9. The patient was subsequently discharged with recommendations for continued outpatient follow-up. Further follow-up visits were planned to monitor the evolution of retinal changes and assess long-term visual outcomes.

## 3. Discussion

Commotio retinae is a well-recognized consequence of blunt ocular trauma, resulting from mechanical disruption of the photoreceptor outer segments and retinal pigment epithelium due to rapid deformation of the globe [1]. Previous studies have shown that, in the absence of additional retinal pathology, commotio retinae often follows a self-limiting course with favorable functional outcomes [2]. Structural alterations involving the outer retinal layers, particularly the photoreceptor inner and outer segment junction, have been demonstrated using optical coherence tomography (OCT) [6,7].

Pediatric cases of commotio retinae have been reported in association with various types of blunt ocular trauma. For example, injuries caused by high-velocity impacts, such as baseball-related trauma, have been described, typically demonstrating transient retinal whitening and reversible outer retinal changes on OCT [2]. Similarly, cases following blunt trauma during physical struggle have shown more severe macular involvement, including disruption of the photoreceptor layer, increased retinal thickness, and, in some instances, persistent structural abnormalities affecting visual recovery [8].

However, despite the variety of reported mechanisms, we did not identify previously published pediatric cases of commotio retinae with macular involvement specifically following a fall from a horse.

Visual outcomes after blunt ocular trauma are generally favorable. However, recovery may vary depending on the extent and location of retinal involvement [9]. Although visual acuity improved significantly in the present case, subtle outer retinal irregularities persisted on OCT at follow-up. These structural abnormalities may indicate that anatomical recovery is not fully complete and suggest the possibility of subtle functional disturbances that are not detected by standard visual acuity testing, such as reduced contrast sensitivity or paracentral visual field defects. Therefore, longer follow-up, including both structural and functional assessment, may be important to fully evaluate visual outcomes in patients with traumatic macular involvement.

Falls from horses represent a recognized mechanism of high-energy blunt trauma, particularly in pediatric and adolescent populations [5]. Although most equestrian injuries involve the head and extremities, ocular trauma may also occur and lead to vision-threatening complications [5,10]. Falls remain a common cause of ophthalmic trauma in equestrian sports, with many patients experiencing visual impairment [10]. Reports focusing specifically on posterior segment involvement in pediatric equestrian trauma remain limited.

Multimodal retinal imaging played a key role in the diagnosis and follow-up of our patient. Early-phase fluorescein angiography showed central hypofluorescence in the foveal region with surrounding mild mottled hyperfluorescence, without clear vascular abnormalities. OCT enabled detailed visualization of dynamic structural changes, including alterations in retinal contour and ellipsoid zone irregularities. Previous studies have highlighted the value of OCT in detecting retinal alterations following blunt ocular trauma and in monitoring their evolution over time [6,7,11]. Taken together, although individual imaging findings may not be entirely specific, the overall clinical and multimodal imaging presentation supports the diagnosis of post-traumatic macular involvement within the spectrum of commotio retinae.

The optimal management of commotio retinae remains a matter of debate, as no standardized treatment guidelines have been established. In most cases, the condition is self-limiting and managed conservatively with observation alone [2]. However, in selected cases with significant visual impairment or macular involvement, additional therapeutic approaches have been considered. In the present case, systemic corticosteroids and topical NSAIDs were administered with the aim of addressing a possible inflammatory component of post-traumatic retinal injury and limiting secondary tissue damage. The rationale for this approach is based on the hypothesis that inflammation may contribute to photoreceptor dysfunction following blunt trauma. Nevertheless, the role of corticosteroids in commotio retinae remains controversial, and evidence supporting their routine use is limited. Some reports have described functional and anatomical improvement following corticosteroid therapy in traumatic maculopathy; however, these observations are based primarily on isolated case reports and small case series [8]. Therefore, it remains unclear whether the observed improvement is attributable to treatment or reflects the natural course of the condition. Alternative management strategies include careful observation with serial imaging, which remains the standard approach in most patients, as well as, in selected cases, the use of systemic corticosteroids, intravitreal therapy, or other interventions aimed at reducing macular involvement. Given the lack of high-quality evidence, treatment decisions should be individualized based on clinical presentation and severity of retinal changes.

Clinically, this case highlights the importance of early ophthalmic evaluation following blunt ocular trauma, particularly in pediatric patients. It also underscores the role of multimodal imaging in assessing retinal structure and monitoring recovery. Proper safety measures, including rider education and helmet use, may help reduce the incidence and severity of equestrian-related injuries [12,13].

## 4. Conclusions

Given the underestimated risk of ocular trauma in equestrian activities, especially in children and adolescents [14,15], increased awareness and standardized follow-up for patients with commotio retinae are crucial. The promotion of protective equipment, particularly helmets, remains a vital strategy to prevent severe ocular and cranial injuries. Prompt ophthalmic evaluation, detailed imaging, and carefully tailored treatment may influence visual outcomes in trauma-related macular injuries.

## Data Availability

The original contributions presented in this study are included in the article. Further inquiries can be directed to the corresponding author.
